# Genetic basis and imaging findings of neurofibromatosis 1 and other somatic overgrowth disorders

**DOI:** 10.1007/s00256-024-04772-7

**Published:** 2024-09-10

**Authors:** Orsolya Vittay, Joseph Christopher, Sarju G. Mehta, Andoni P. Toms

**Affiliations:** 1 Department of Radiology, Addenbrooke’s Hospital, Hills Road, Cambridge, CB2 2QQ UK; 2 Department of Clinical Genetics, Addenbrooke’s Hospital, Hills Road, Cambridge, CB2 2QQ UK; 3 Academic Department of Medical Genetics, University of Cambridge, Cambridge Biomedical Campus, Cambridge, CB2 0QQ UK; 4 Norwich Medical School, University of East Anglia, Norwich Research Park, Norfolk, NR4 7TJ UK; 5 Department of Radiology, Norfolk & Norwich University Hospital, Colney Lane, Norwich, NR4 7UB UK

**Keywords:** Neurofibromatosis, Somatic overgrowth, Focal gigantism

## Abstract

Somatic overgrowth disorders comprise a wide range of rare conditions that present with focal enlargement of one or more tissue types. The PI3K-AKT-mTOR pathway is a signalling pathway that induces angiogenesis and cell proliferation, and is one of the most commonly overactivated signalling pathways in cancer. The PI3K-AKT-mTOR pathway can be up-regulated by genetic variants that code for proteins in this pathway, or down-regulated by proteins that inhibit the pathway. Mosaic genetic variations can result in cells that proliferate excessively in specific anatomical locations. The *PIK3CA*-related overgrowth spectrum (PROS) disorders include CLOVES syndrome, macrodystrophia lipomatosa, and Klippel-Trenaunay syndrome among many. The neurofibromatosis type 1 (NF1) gene encodes neurofibromin which down-regulates the PI3K-AKT-mTOR pathway. Thousands of pathological variants in the *NF1* gene have been described which can result in lower-than-normal levels of neurofibromin and therefore up-regulation of the PI3K-AKT-mTOR pathway promoting cellular overgrowth. Somatic overgrowth is a rare presentation in NF1 with a wide range of clinical and radiological presentations. Hypertrophy of all ectodermal and mesodermal elements has been described in NF1 including bone, muscle, fat, nerve, lymphatics, arteries and veins, and skin. The shared signalling pathway for cellular overgrowth means that these radiological appearances can overlap with other conditions in the *PIK3CA*-related overgrowth spectrum. The aim of this review is to describe the genetic basis for the radiological features of NF1 and in particular compare the appearances of the somatic overgrowth disorders in NF1 with other conditions in the *PIK3CA*-related overgrowth spectrum.

## Introduction

Limb hypertrophy in neurofibromatosis type 1 (NF1) is rare and the clinical presentation is highly variable. The condition may affect a single digit or a whole limb. The usual phenotype comprises disordered overgrowth primarily of bone and neural tissue with plexiform or diffuse neurofibromas predominating, but other tissues are also involved. Mosaic *NF-1* variants can lead to mesodermal dysplasia that also result in overgrowth of fat, fibrous, and vascular elements. In these cases, it can be difficult to differentiate the imaging appearances from other causes of somatic overgrowth. The first aim of this paper is to outline the common pathway that mediates the majority of somatic overgrowth disorders to explain the overlap in imaging phenotypes. The second is to describe the range of imaging findings in limb hypertrophy in NF1 and compare these with other disorders with similar appearances.

## PI3K/AKT/mTOR pathway

**Fig. 1:**
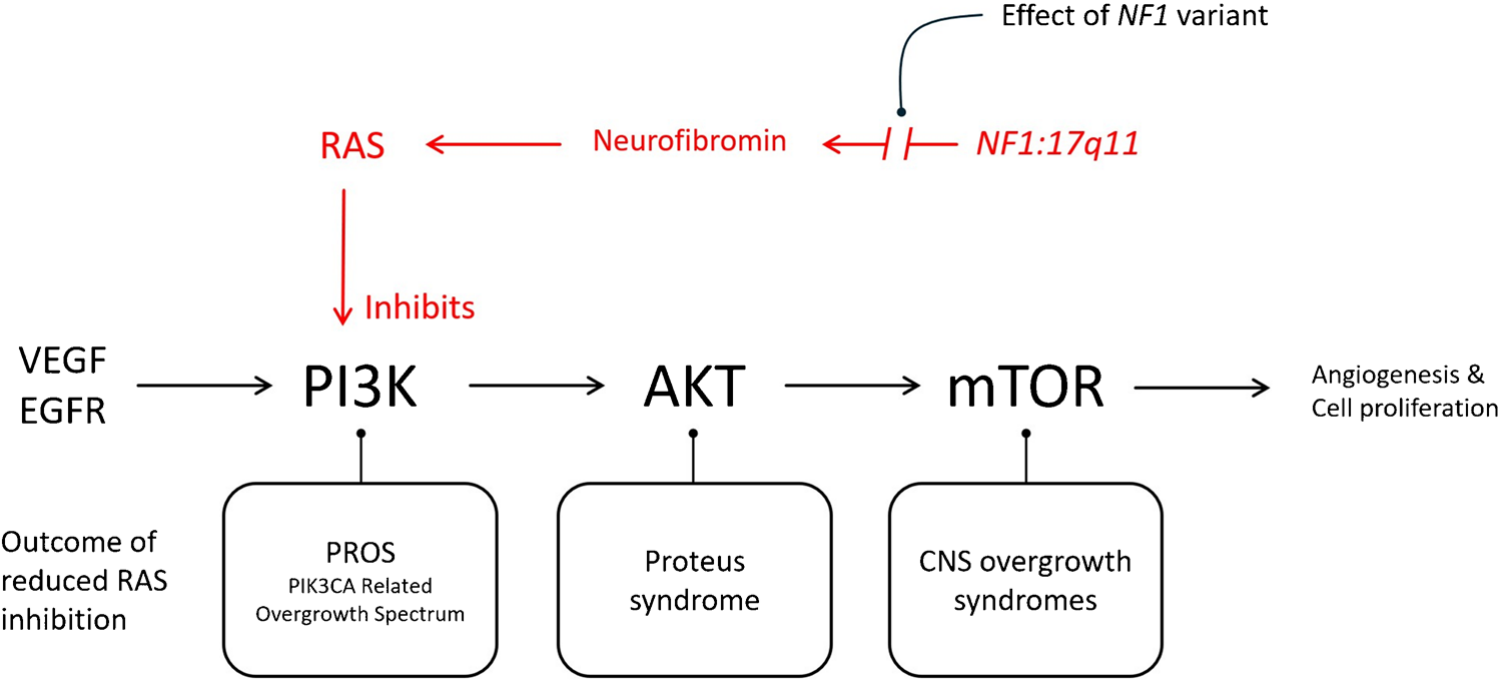
Schematic diagram of the influence of reduced levels of RAS in NF1 on the PI3K-AKT-mTOR pathway with some of the somatic overgrowth disorders that result from the uninhibited pathway. The PIK3CA-related overgrowth spectrum (PROS) includes macrodystrophia lipomatosa, Klippel-Trenaunay syndrome, CLOVES syndrome, fibroadipose vascular anomaly, macrodactyly, and lipomatosis of nerve among others

The phosphoinositide 3-kinase (PI3K)/protein kinase B (AKT)/mechanistic target of rapamycin (mTOR) pathway is a major intracellular signalling pathway that leads to cell proliferation [1] (Fig. 1). mTOR is a serine-threonine kinase that acts as a key regulator of cell growth, and it is disrupted in many disorders of cell proliferation including malignancies [2]. The significant role of the PI3K/AKT/mTOR pathway in the pathogenesis of many cancers means that it is increasingly being targeted in the development of novel oncological therapies. Through its effects on cell proliferation, it also plays an important role in tissue development and angiogenesis and is therefore implicated in the pathophysiology of multiple overgrowth syndromes [3].

The molecular basis of various limb overgrowth syndromes is becoming increasingly well understood. Growth factors such as vascular endothelial growth factor (VEGF) and epidermal growth factor receptor (EGFR) mediate cellular growth by upregulating the PI3K enzyme early in the PI3K/AKT/mTOR pathway. The *PIK3CA* gene encodes the catalytic subunit of the PI3K enzyme [3]. Therefore, somatic variants in *PIK3CA* may lead to a variety of types of overgrowth, including varying degrees of vascular overgrowth and malformation, or both, as well as limb hypertrophy [4]. These overgrowth syndromes are collectively known as the *PIK3CA*-related overgrowth spectrum (PROS) and include Klippel-Trénaunay syndrome (KTS), CLOVES syndrome, and fibroadipose vascular anomaly (FAVA). There is often overlap in the phenotype of these conditions making diagnosis difficult.

Further downstream in the pathway, somatic variants in *AKT-1* (encoding protein kinase B or AKT), which regulates cell survival [5], lead to the development of the soft tissue and dermatological findings pathognomonic of Proteus syndrome, particularly cerebriform connective tissue naevi. Certain phenotypes of Proteus syndrome also include asymmetrical limb overgrowth and low-flow vascular malformations [3].

NF1 is an autosomal dominant genetic disorder with complete penetrance and variable expression although approximately half of cases may be de novo and therefore without a family history [6]. It is classically characterised by the presence of multiple peripheral neurofibromas [7], but may also feature skeletal dysplasia, and in a minority of cases, limb overgrowth. The *neurofibromin-1* (*NF-1*) gene is located on the long arm of chromosome 17 (17q11.2) and functions as a tumour suppressor gene [8, 9]. *NF-1* encodes neurofibromin, a cytoplasmic protein predominantly expressed in neurons, non-myelinating Schwann cells, and oligodendrocytes [10], which acts partially as a Ras-GTPase-activating protein (RasGAP), thus negatively regulating the Ras pathway [2, 11]. When neurofibromin is lost or mutated, Ras and its downstream signalling intermediates are hyperactivated, transmitting the Ras growth signal to PI3K, causing AKT-dependent phosphorylation, and finally causing the aberrant activation of mTOR. The mTOR molecule becomes constitutively up-regulated, initiating uncontrolled cellular growth and angiogenesis. This ultimately leads to the development of a variety of benign and malignant tumours seen in NF1 [3, 7]. It may also result in focal limb hypertrophy because of mosaic variants [11]. There are many different *NF-1* variants each with different levels of disinhibition of the RAS pathway.

## Mosaicism

**Fig. 2:**
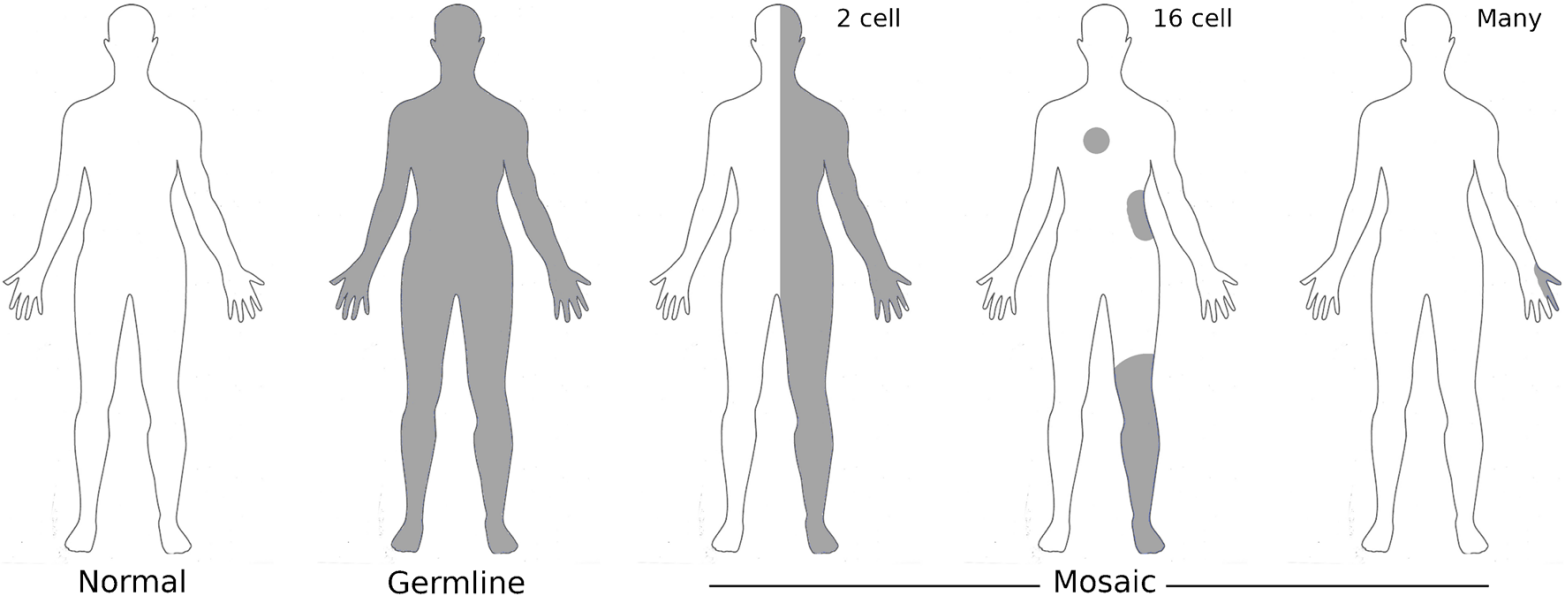
Diagrammatic representation of the musculoskeletal phenotype of a variety of mosaic mutations that may occur early in formation of the blastocyst to later in limb formation

Mosaicism is the result of two genetically separate cell lines in a single individual resulting from the acquisition of a post-zygotic gene variant [12] (Fig. 2). Mosaicism can present as segmental, generalised, or the very rare gonadal forms [12, 13]. In segmental mosaicism, the features of NF1 are restricted to one or more segments of the body and are responsible for regional skin pigmentation, tumour growth [12], and limb hypertrophy. It is the combination of the variety of *NF-1* variants, and their variable effect on the normal inhibition of the RAS pathway, epigenetic modifiers, and the stage of post-zygotic cell division that produces the heterogeneous phenotype of NF1 in the musculoskeletal system.

## Neurofibromas

Neurofibromas are classified as a type of benign hamartoma, belonging to the group of benign peripheral nerve sheath tumours, according to the WHO classification of tumours of the nervous system [14]. The cells driving the formation of the tumour are Schwann cells that contain variants in the *NF-1* gene that disinhibit the local RAS pathway causing cell proliferation, but this cell proliferation is not confined to Schwann cells [15]. The neurofibroma hamartomas also contain fibroblasts, mast cells, blood vessels, and an extracellular matrix of mucin. Only the Schwann cells have loss of heterozygosity at the *NF-1* locus [16, 17]. The other cellular components are recruited by microenvironment reprogramming as a result of the PI3K-AKT-mTOR pathway downstream effectors [18]. They may be of widely varying sizes, may develop in several locations, and may occur with or without the presence of NF1 [19].

A formal clinical classification system for neurofibroma subtypes is currently lacking [13]. However, neurofibromas can be organised into three broad types: localised, plexiform, and diffuse, with further subtypes within these categories.

Localised neurofibromas are confined to a single nerve and may be subdivided into cutaneous or intraneural neurofibromas. They commonly occur outside the setting of NF1. Cutaneous neurofibromas are the most common subtype of neurofibroma and normally arise from a single cutaneous nerve [13]. These tumours normally present from adolescent life onwards, can present anywhere on the body, and have virtually no risk of malignant transformation. Two or more neurofibromas of any type are part of the diagnostic criteria for NF1 [20], although NF1 patients may have no neurofibromas, or up to many thousands. A comprehensive classification system for further subtypes of cutaneous neurofibromas has been proposed [21].

Intraneural neurofibromas are classically characterised by segmental or fusiform nerve enlargement and may occur anywhere in the body. Neurofibromas may also affect the subcutaneous tissues and thoracic and abdominal organs, and may rarely cause serious complications secondary to their anatomical position and resulting mass effect [22].

**Fig. 3:**
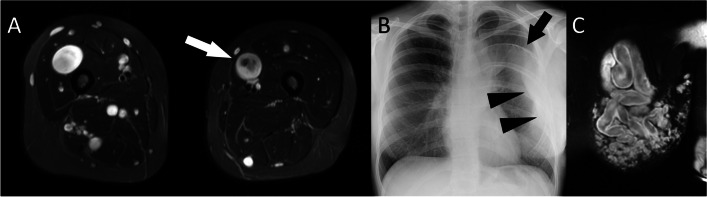
**A** Axial T2 fat-saturated MR image through both thighs in a patient with NF1 demonstrated multiple neurofibromas characterised by hyperintense signal in the periphery with hypointense signal centrally. **B** A PA chest radiograph in a patient with a plexiform neurofibroma demonstrates a soft tissue mass (arrowheads) and a scalloping of the left 5th rib. **C** A coronal T2W fat-saturated MR image through the left chest wall demonstrates a complex tubular mass with signal characteristics typical of a neurofibroma. The shape of the left 5th rib may be the result of focal dysplasia rather than a direct pressure effect

Understanding the imaging findings of neurofibromas can be directly related to their composition. Neurofibromas containing a mucinous extracellular matrix which is rich in water demonstrate a characteristic homogeneous hyperintense signal on T2-weighted magnetic resonance imaging (MRI). When they contain a dense central collagenous component that replaces the mucinous water, the T2 signal is reduced centrally but maintained in the periphery of the lesion creating the classic ‘target’ sign (Fig. 3).

Plexiform neurofibromas (PN) are a complex type of neurofibroma involving multiple nerve bundles or fascicles, and may also occur anywhere in the body [19]. In contrast with localised neurofibromas, PNs are commonly congenital and typically grow most rapidly in the first ten years of life. They also possess the potential for malignant transformation into a malignant peripheral nerve sheath tumour (MPNST), which occurs in approximately 8–12% of NF1 patients [12]. PNs are considered virtually pathognomonic for NF1, although rare cases of these neurofibromas in the absence of NF1 have been reported [23]. They may be associated with modelling abnormalities of adjacent bones giving the impression of a mass effect; however, since the osseous modelling abnormalities in NF1 can be seen without adjacent neurofibromas, it is more likely that any nearby skeletal deformity is part of a localised combined bone and soft tissue dysplasia.

The classic MR imaging description is of a complex lobulated mass of tissue sometimes described as a ‘bag of worms’ with similar signal characteristics to isolated neurofibromas: hyperintense signal on T2-weighted imaging, hypointense signal on T1-weighted imaging when compared to the surrounding muscle, and a ‘target sign’ (Fig. 3) [24, 25]. In cases where MPNST is suspected, contrast-enhanced MRI may be helpful in demonstrating perilesional enhancement [26].

**Fig. 4:**
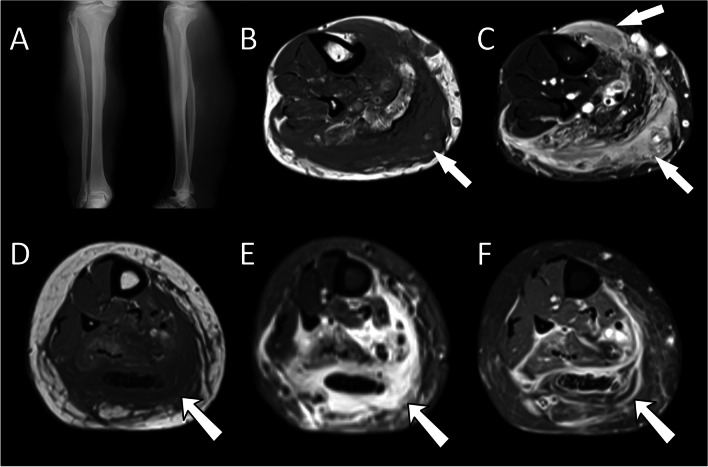
**A** Anteroposterior and lateral radiographs of the tibia and fibula in a patient with neurofibromatosis type 1 presenting with an enlarged calf comprising a diffuse plexiform neurofibroma and dysplasia of both the tibia and fibula. Axial T1W (**A**) and T2 fat-saturated (**B**) images through the calf reveal a diffuse seemingly “infiltrative” intermediate T1w and hyperintense T2W soft tissue lesion that does not respect anatomical compartments (arrows). Biopsy of the lesion demonstrated a diffuse plexiform neurofibroma that remained stable on MR imaging over a period of 12 years

**Fig. 5:**
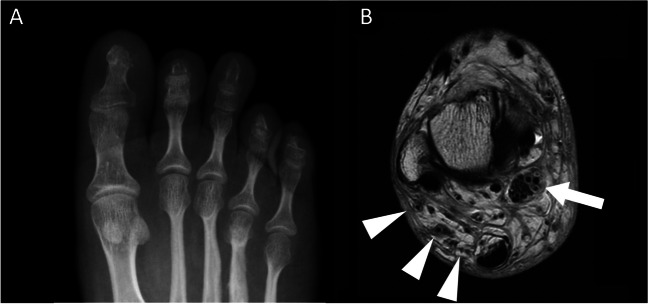
Axial T1W (**A**), fat-saturated T2W (**B**), and fat-saturated T1W after intravenous gadolinium (**C**). Within the diffuse plexiform neurofibroma are areas of hyperintense T2W signal that fail to enhance (arrows) indicating the presence of myxoid tissue in a gelatinous variant plexiform neurofibroma

Diffuse neurofibromas are a rare subtype of neurofibroma, characterised by a diffusely infiltrating lesion, comprising all the cellular and extracellular elements of a neurofibroma, which replaces other mesenchymal tissues such as muscle and bone, and which ignores compartmental boundaries [27]. These lesions present in children and young adults and may occur in the absence of other stigmata of NF1 [27, 28]. Given that the histological composition of the lesion is the same as other types of neurofibroma, the MRI signal characteristics are identical. However, due to the infiltrative nature of the neurofibroma, these lesions may be missed or mistaken for oedema, mesenchymal tumours, or lymphangiomas as they extend along the fascial planes [29] (Fig. 4). MRI, with intravenous gadolinium, demonstrates enhancement of the cellular and vascular component of the lesion, with a lack of enhancement of the mucinous component, which can help differentiate it from other inflammatory or infiltrative diseases (Fig. 5).

## Skeletal abnormalities in NF1

**Fig. 6:**
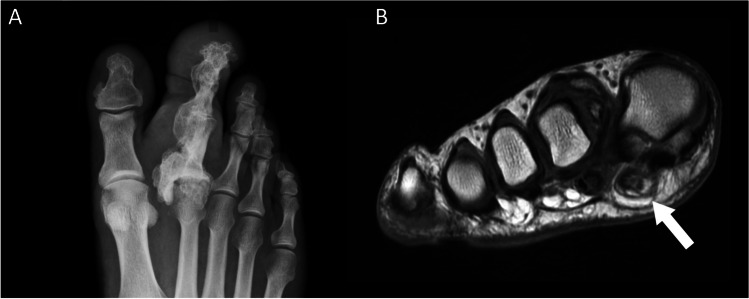
Radiographs of the right foot in the patients with (**A**) NF1 and (**B**) macrodystrophia lipomatosa with focal gigantism of the (A) third and (B) second toes. The patient with NF1 (A) has a long third toe with little soft tissue hypertrophy but they do also have a dysplasia of the 4th metacarpal. The patient with macrodystrophia lipomatosa has hypertrophy of the bone and overlying soft tissues in a sclerotomal distribution along the medial digital nerve

Various abnormalities of the appendicular skeleton in patients with NF1 have been described. These include bone hypertrophy and dystrophy [30]. Dystrophy of the appendicular skeleton manifests as bowing of the long bones, most commonly the tibiae, pseudoarthrosis, distorted bone growth, and local bony erosions (Fig. 6) [31, 32]. Rarer skeletal manifestations include ossifying subperiosteal haematomas and the subsequent formation of so-called ‘cortical bone cysts’ [33]. These are often attributed to pressure and mass effect from neighbouring neurofibromas [28; however, skeletal dysplasia can be independent of local neurofibromas [30].

## Limb overgrowth in NF1

Somatic limb overgrowth in NF1 presents with combinations of the neurofibromatous hamartomas and bone dysplasias described above, in combination with variable hypertrophy of fat and blood vessels. This combination of findings is often described as elephantiasis neuromatosa, and it is a rare manifestation of NF1 with a long historical background [34]. It is thought that most of these cases occur because of mosaic variants in the NF1 gene, where a single limb or digit is affected, causing localised hypertrophy of the affected extremity (Fig. 6A). The extent of hypertrophy may often be substantial, often necessitating surgical debulking if not amputation of the affected limb in extreme cases. The lower limb is the most affected site where it is present in 46% of subjects reported in the scientific literature. Other sites including the head and neck, the trunk, and, more rarely, the upper extremity can also be affected [35, 36].

Several reports in the literature describe limb overgrowth in NF1 as being synonymous with a massive neurofibromatous lesion or multiple smaller neurofibromas [29, 35, 36]. However, recent improvements in understanding of NF1 pathophysiology indicate that hypertrophy is likely to be secondary to mesodermal or ectodermal dysplasia [37], or both, and therefore may affect many subsequent cell lines including bone, nerve, blood vessels, fat, and lymphatics. This is in common with other causes of limb overgrowth.

**Fig. 7:**
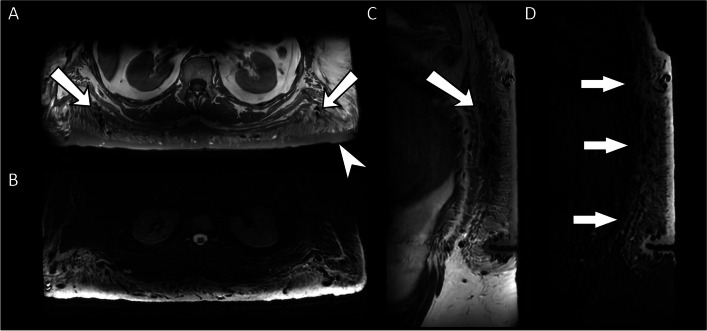
**A** Coronal T1W MR image in a patient with macrodystrophia lipomatosa demonstrating lipofibromatous hamartoma of the lateral digital nerve of the great toe (arrow). **B** An axial PD-weighted MR of the ankle in a patient with neurofibromatosis type 1 demonstrates similar thickening of the neural bundles and Schwann cells of the posterior tibial nerve (arrow) as well as multiple extra vessels (arrowheads) in the hypertrophied fat of Kager’s fat pad

Macrodystrophia lipomatosa is a rare disorder of localised limb overgrowth [38] which typically presents with one or more enlarged digits, usually in the distribution of the median (upper limb) and plantar nerves (lower limb) [39] (Figs. 6B and 7A). Patients may present with functional problems such as an inability to grip objects, symptoms secondary to vascular and neural compromise, or simply dissatisfaction with cosmetic appearance [40].

Histology demonstrates hypertrophy of the fatty adipose tissues intermixed within a fibrous tissue matrix, with involvement of the underlying tissues including the subcutaneous tissues, nerve sheaths, muscles, periosteum, and bone marrow [39]. Plain radiographs demonstrate corresponding hypertrophy of the affected bones with radiolucencies within the surrounding soft tissues in keeping with hypertrophied fat (Fig. 6B). MRI demonstrates hypertrophic fat with identical signal characteristics to normal subcutaneous fat [41]. Hypertrophied neural bundles with interspersed fat may indicate a concomitant lipofibromatous hamartoma (LFH) of nerve (Fig. 7A), commonly present when macrodystrophia lipomatosa affects a nerve associated with a distal extremity [42, 43].

**Fig. 8:**
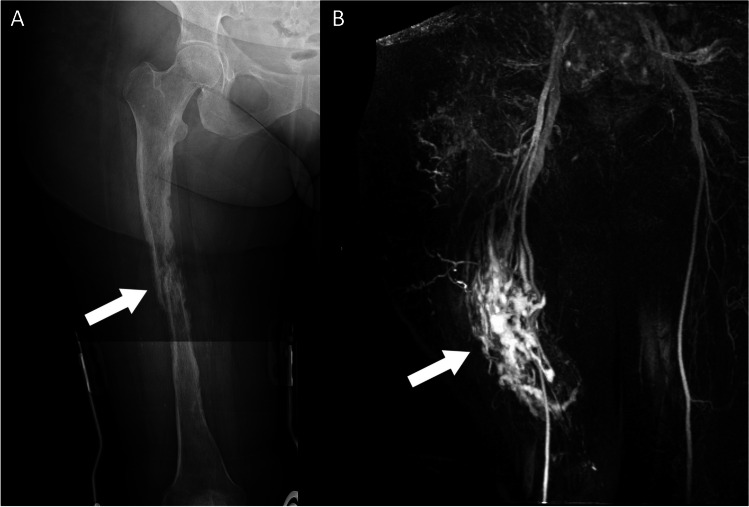
Axial (**A** and **B**) and sagittal (**C** and **D**) T1 and T2 fat-saturated MR images of the posterior thoracoabdominal wall in a patient with NF1 demonstrating hypertrophy of the skin (arrowhead) and subcutaneous fat, which is hyperintense on the T1W sequences (**A** and **C**), interspersed with a biopsy-proven diffuse plexiform neurofibroma that is hyperintense of the fluid sensitive sequences (**D,** white arrows). The whole area contains multiple convoluted flow voids (**A** and **C**, arrows)

It is often difficult to differentiate macrodystrophia lipomatosa from NF1 due to similarities in clinical presentation. Important clinical differences include the possibility of bilateral involvement of the digits in NF1, commonly non-contiguous hypertrophy of the extremity in NF1, and cessation of growth associated with macrodystrophia lipomatosa by puberty [40]. It has been suggested that focal gigantism with fibrofatty tissue masses on MRI is so characteristic of macrodystrophia lipomatosa that no other diagnosis need be considered [41]. While fat hypertrophy on MRI does not appear to be a common finding in limb hypertrophy associated with NF1, it has been clearly described and therefore it is not a specific discriminator for the two conditions [44] (Fig. 8).

While diffuse enlargement of peripheral nerves is more commonly associated with macrodystrophia lipomatosa, it has been described once in NF1 when marked vagal nerve hypertrophy in NF1 has been reported as an incidental finding prior to vagal nerve stimulator implantation [45]. Sonography demonstrated marked homogeneous enlargement of the vagus nerve in the axial and sagittal planes in keeping with isolated hypertrophy of the affected nerve fibres. These appearances are not completely typical of LFH in which the sonographic findings are more likely to demonstrate hypoechoic cable-like neural bundles separated by hyperechoic fat [42]. However, in our experience, appearances indistinguishable from LFH can be seen on MRI in patients with NF1 (Fig. 7).

Vascular and lymphatic proliferation are rare complications of NF1. They are more usually associated with Klippel-Trénaunay syndrome (KTS) and Gorham’s disease, both of which may present with limb overgrowth.

**Fig. 9:**
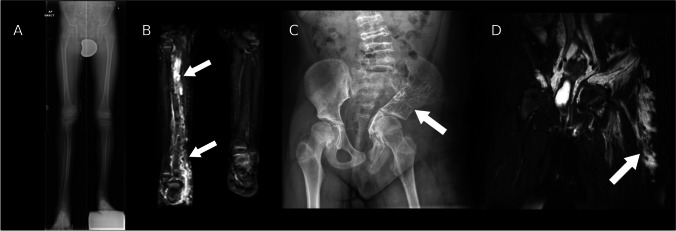
Stitched anteroposterior radiographs of the right femur (**A**) in a 52-year-old woman who presented to the emergency department with a spontaneous mid-diaphyseal femoral fracture on a background of dysplastic bone. An MR angiogram (**B**) revealed a complex arteriovenous malformation part of Klippel-Trenaunay-Weber syndrome

KTS is a rare syndrome with an estimated incidence of 0.02–0.05 per 1000 live births characterised by a clinical triad of cutaneous port-wine stain (capillary haemangioma), hypertrophy of an extremity, and a complex vascular malformation, with two out of three features required for the diagnosis [46–48]. KTS is a subset of PROS, and therefore is likely to have features in common with other overgrowth syndromes. Lymphatic malformations are estimated to occur in approximately 11% of patients, while limb hypertrophy occurs in up to 67%, with close to 90% of these affecting the lower limb [49]. The underlying bone of the affected limb is often dysplastic, which may lead to pathological fractures (Fig. 9A).

Gorham’s disease, also known as lymphangiomatosis, is characterised by vascular and lymphatic proliferation into bone, resulting in progressive osteolysis of the underlying bone [50, 51], and giving rise to the description ‘disappearing bone disease’ [52]. Radiological features include multiple lytic lesions of the bones, occasionally dramatic in their extent, most noted in the vertebrae, pelvis, and ribs, which may lead to pathological fractures [53]. Lymphangiomatosis may also affect multiple internal organs such as the spleen and the lungs, causing localised parenchymal lesions and subsequent organ dysfunction. Lymphatic proliferation may also extend to the soft tissues, leading to hypertrophy of the affected limb secondary to soft tissue lymphangiomatosis (Fig. 10C).

There are limited case reports of vascular proliferation in the scientific literature. There is one case of mesenteric vascular involvement of NF1 causing a protein-losing enteropathy [54]. A second case reports markedly dilated and tortuous lymphatic vessels on lymphangiography in the left leg of a young girl with NF1 which was interpreted as being caused by occlusion of the lymphatic channels secondary to plexiform neurofibroma growth [55]. To add to these, we present a third case of lymphatic hypertrophy diagnosed on ultrasound and MRI in a child with limb overgrowth in NF1 (Fig. 10). While lymphatic dilation and proliferation in NF1 may be secondary to mass effect from neurofibromas, our current understanding of the common pathways that mediate tissue growth suggests that lymphatic proliferation in NF1 may be the result of mesodermal dysplasia.

## Conclusion

Somatic overgrowth disorders in NF1 are rare and varied. Mosaic variants in the NF1 gene cause disordered regulation of tissue growth, mediated by the PI3K-AKT-mTOR pathway, localised to specific parts of the appendicular skeleton. The result is a mesodermal and ectodermal dysplasia with the potential for overgrowth of all tissue elements. While there are characteristic imaging phenotypes in NF1, there is overlap with the imaging appearances of other somatic overgrowth conditions and therefore NF1 genetic testing ideally from the affected tissue is essential for diagnosis.
